# A new age in AquaMedicine: unconventional approach in studying aquatic diseases

**DOI:** 10.1186/s12917-018-1501-5

**Published:** 2018-06-08

**Authors:** Michael Gotesman, Simon Menanteau-Ledouble, Mona Saleh, Sven M. Bergmann, Mansour El-Matbouli

**Affiliations:** 1 Department of Biology, New York City College of Technology of the City University of New York, Brooklyn, New York, USA; 2 Clinical Division of Fish Medicine, Department for Farm Animals and Veterinary Public Health, University of Veterinary Medicine, Veterinärplatz 1, 1210 Vienna, Austria; 3 Institute of Infectology, Friedrich-Loffler-Institut (FLI), Federal Research Institute for Animal Health, Greifswald-Insel Riems, Germany

**Keywords:** Aquaculture, Virus, Bacteria, Nanotechnology, RNAi, CRISPR/Cas

## Abstract

**Background:**

Marine and aquaculture industries are important sectors of the food production and global trade. Unfortunately, the fish food industry is challenged with a plethora of infectious pathogens. The freshwater and marine fish communities are rapidly incorporating novel and most up to date techniques for detection, characterization and treatment strategies. Rapid detection of infectious diseases is important in preventing large disease outbreaks.

**Main text:**

One hundred forty-six articles including reviews papers were analyzed and their conclusions evaluated in the present paper. This allowed us to describe the most recent development research regarding the control of diseases in the aquatic environment as well as promising avenues that may result in beneficial developments. For the characterization of diseases, traditional sequencing and histological based methods have been augmented with transcriptional and proteomic studies. Recent studies have demonstrated that transcriptional based approaches using qPCR are often synergistic to expression based studies that rely on proteomic-based techniques to better understand pathogen-host interactions. Preventative therapies that rely on prophylactics such as vaccination with protein antigens or attenuated viruses are not always feasible and therefore, the development of therapies based on small nucleotide based medicine is on the horizon. Of those, RNAi or CRISPR/Cas- based therapies show great promise in combating various types of diseases caused by viral and parasitic agents that effect aquatic and fish medicine.

**Conclusions:**

In our modern times, when the marine industry has become so vital for feed and economic stability, even the most extreme alternative treatment strategies such as the use of small molecules or even the use of disease to control invasive species populations should be considered.

## Background

### Fish and aquatic industry

The global annual per capita fish consumption of fish was 20.1 kg for the year 2013; with an average consumption of 26.8 kg in industrialized countries and 18.1 kg in developing countries, respectively [1]. Fish protein accounted for up to 20% or more of the total animal protein consumed in low-income food-deficient countries and around 17% globally in the year 2013 [1]. In addition to producing a critical part of nutrition, food production through the marine industry also represents a major form of employment which harvested nearly 160 billion US dollars in 2014 and employed 56.6 million people [1]. Of the total 167.2 million tonnes of food products that the marine industry produced in 2014, the aquaculture accounted for 44.1% (73.8 million tonnes) production [1]. Carp (*Cyprinidae*) makes up a significant portion of the total freshwater cultured fish supply and is an especially important food in China and the remainder of East Asia, which produced 61.3 and 26.7% of cultured carp, respectively in 2010 [1, 2]. Salmon and shrimp are considered high-value species that are heavily traded and are also significant members of the aquaculture industry [1].

### Viral diseases of fish and crustaceans in the aquatic industry

Diseases of aquatic products, such as viral infection of aquatic animals, have become more problematic and are causing significant economical losses to the aquaculture industry [3–7]. The most serious viruses affecting cyprinid fish including koi and common carp (*Cyprinus carpio L.*) are: 1) Cyprinid herpesvirus 3 (CyHV-3), a member of the *Alloherpesviridae* family of viruses, which is the aetiological agent of a highly contagious disease termed Koi herpesvirus disease (KHVD) and 2) spring viraemia of carp virus (SVCV), which is a member of the *Rhabdoviridae* family of viruses. In addition, outbreaks of KHVD and SVC in cultured common carp caused significant economic losses in recent years. KHVD has been a major research topic in aquatic medicine and has been listed as a notifiable disease in Germany since 2005, in England and by the World Organization of Animal Health (OIE) since 2007 [8–13]. Similarly, the SVC is a major topic of aquatic research and is listed as a notifiable disease in the USA, and has been listed by the OIE since 1997 [14, 15]. Hemorrhagic septicemia virus (VHSV) is another pathogenic member of *Rhabdoviridae* known to infect northern pike, *Esox lucius* fry [16]. Members of the *Rhabdoviridae* family of viruses that code for the non-virion protein (NV) are subtyped into their own genus termed *Novirhabdovirus;* such as infectious hematopoietic necrosis virus (IHNV), which causes an OIE notifiable disease and is an economically important in a wide variety of salmonid species [17]. Infectious salmon anemia virus (ISAV) the causative agent of the ISA and White Spot Syndrome Virus (WSSV) the causative agent of White Spot Disease are of major economic importance in the respective salmon and crustacean aquaculture sector [18, 19]. *Iridoviridae* comprises a family of double stranded DNA virus that infect a wide variety of invertebrate and marine organisms, such as the genus *Megalocytivirus*, represented by red sea bream iridovirus (RSIV) [20, 21]. *Ranavirus,* another representative genus in the *Iridovirdae* family, is a global emergent pathogen capable of infecting fish, amphibians, and reptiles in both captive and wild animals causing hemorrhagic disease [22]. *Betanodaviruses,* which are non-enveloped single stranded RNA viruses, comprise an additional important family of viruses impacting the aquaculture industry [23, 24].

### Bacterial pathogens

**Fig. 1:**
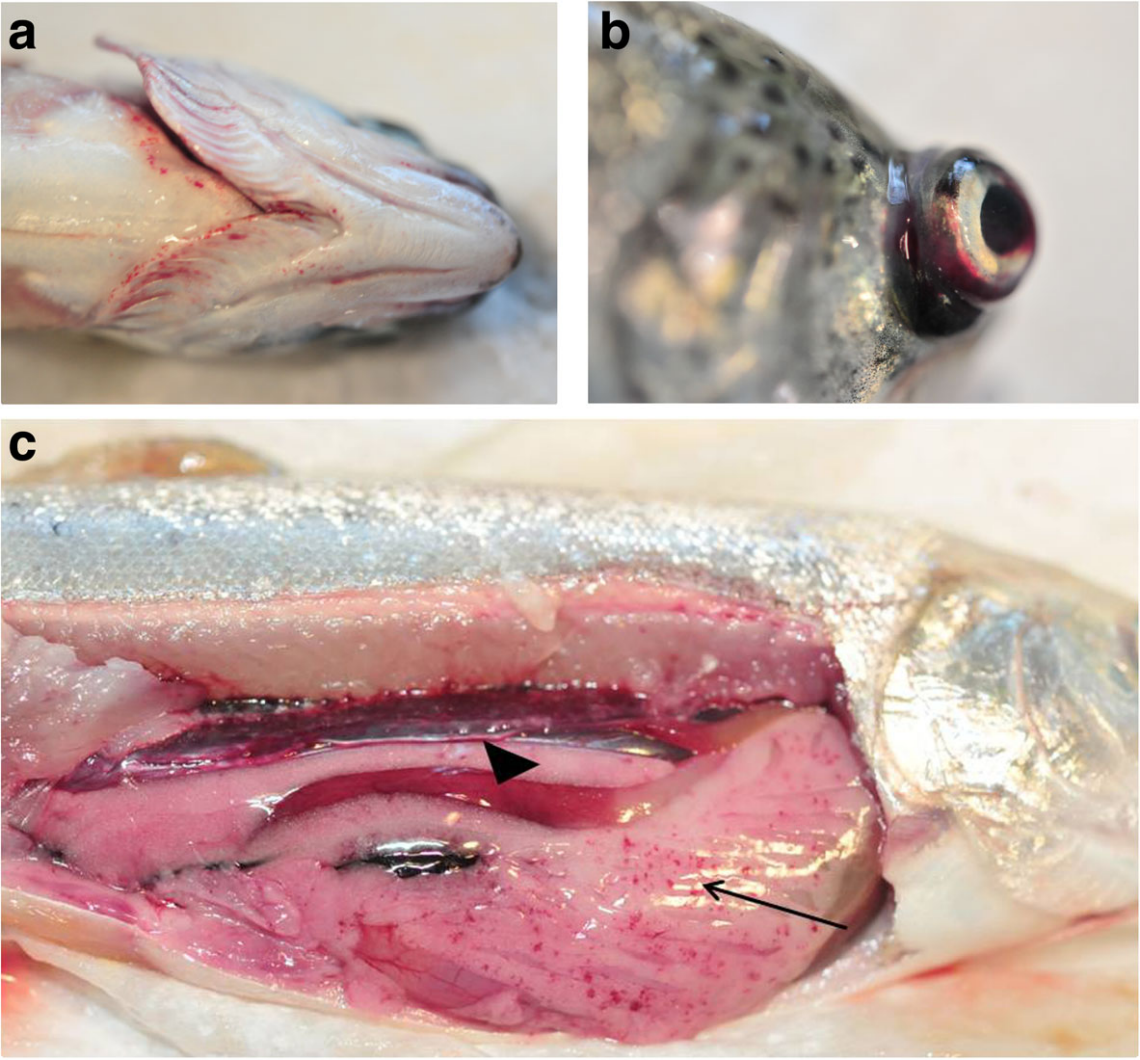
Clinical signs in a rainbow trout (*Oncorhynchus mykiss*) infected with *Yersinia ruckeri* (from personal archives, previously unpublished). **a** Petechia and haemorrhages in the oral region; **b** Exophtalmia; **c** Large arrows- blood in the intestinal track and Small arrow- petechia in the visceral tissue

Among the most note-worthy bacterial aquatic pathogens is the warmwater bacterium *Aeromonas hydrophila* as well as its cold water relative *Aeromonas salmonicida*, which infect a variety of fish species in both the freshwater and marine environment. Among these susceptible fish species, tilapia, cyprinid [25, 26] and salmonid (salmon and trout) fish [26] are of particular economic importance. A bacterium that has mostly been studied in infecting salmonid is the enterobacterium *Yersinia ruckeri*, causative agent of then enteric redmouth disease, salmonid which has been associated with haemorrhages and petechial lesions in infected fish (Fig. 1; from personal archives) [27–29]. Moreover, the enterobacterium *Edwardsiella ictaluri* is considered one of the most important bacterial pathogen affecting the culture of catfish, in particular the channel catfish *Ictalurus punctatus* in the Southern United States [26] and, as it has been more recently reported, the striped catfish *Pangasianodon hypophthalmus* in Vietnam [30, 31]. In shrimp, bacterial infections are mostly linked to bacteria of the *Vibrio* family, in particularly to *Vibrio parahaemolyticus* which has recently been linked to an emergent disease termed acute hepatopancreatic necrosis disease [32].

### Parasites

Parasitic diseases are often associated with more chronic diseases that can cause a sustained loss of productivity over the whole production cycle, and therefore the economical impact of these diseases can often be very significant. Among the most prominent parasitical diseases are the ones caused by myxozoans parasites such as *Myxobolus cerebralis* and *Tetracapsuloides bryosalmonae*, the causative agents of whirling disease and proliferative kidney diseases in salmonids, respectively [33, 34]. Ciliates are common inhabitant of both the freshwater and marine environment. Among these species, *Ichthyophthirius multifiliis* (often shortened to “Ich”) is associated with high levels of mortality (up to 100%) in freshwater fish [35]. This external parasite has a life cycle comprised of three stages, including a trophonts stage during whith it feeds on the fish skin where it forms white circular lesions that leads to the disease colloquial name of “white spot disease” [35–37]. While *I. multifiliis* is limited to freshwater, another ciliated, *Cryptocaryon irritans* is found is saltwater that causes a very similar condition, sometime also referred to as “white spot disease” or “marine ich” [38].

In molluscs, two parasites are particularly noteworthy: *Marteilia refringens* and *Bonamia ostreae* which affects the European flat oyster *Ostrea edulis*. The impact of these parasites has led to the introduction of more resistant species of oyster termed *Crassostrea gigas* to replace *Ostrea edulis* as the most commonly cultivated oyster species. This manuscript will review some of the newest approaches used to study aquatic diseases, in terms of detection characterization and possible treatment strategies.

## Bio diagnostics/characterization

### Rapid detection/nanotechnology

**Fig. 2:**
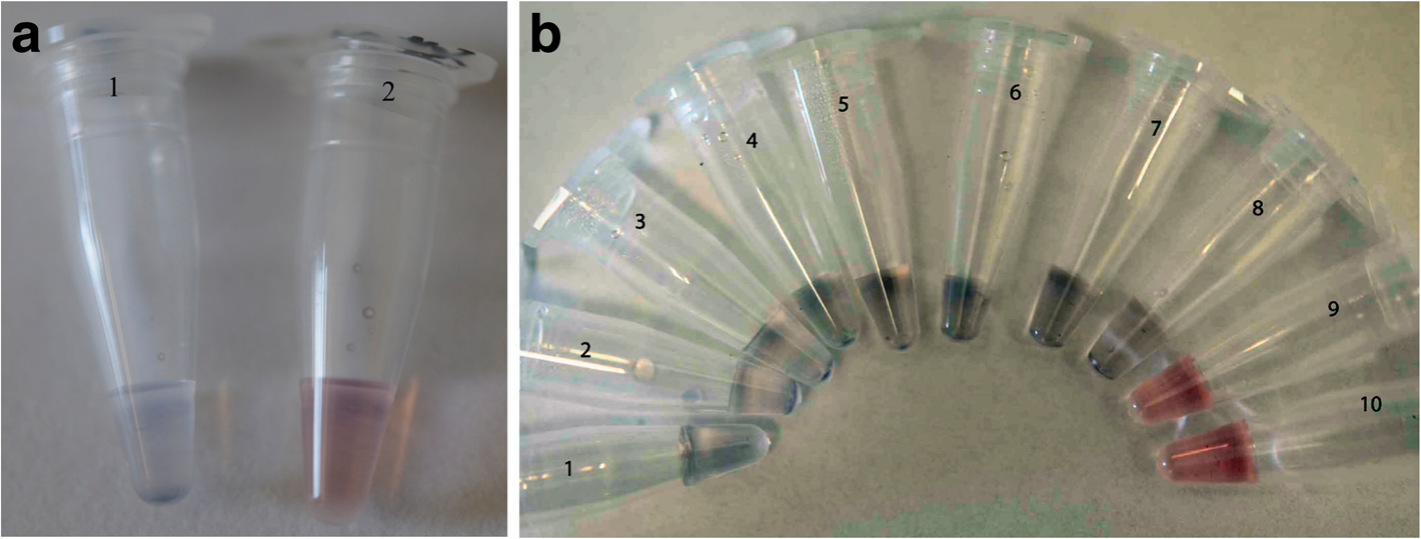
Application of nanoparticles for the diagnostic of spring viraemia of carp virus. **a** Unmodified gold nanoparticles (AuNPs) for the colorimetric detection of spring viraemia of carp virus (SVCV)-RNA (from Saleh et al. [52], figure subject to copyright and r*eproduced with permission* from Springer Nature). Tube 1: Positive SVCV-RNA sample (blue color). Tube 2: No template control (red color). Each tube contained 5 μl of sample, 1.8 μM of primer and 0.1 M NaCl. **b** Serial dilution of SVC-RNA (Tubes 1 to 10 contain 10^5^, 10^4^, 10^3^, 10^2^, 10, 10^–1^, 10^–2^, 10^–3^ 10^–4^ and 10^–5^ TCID_50_ ml ^− 1^ SVC-RNA respectively) showing the sensitivity limit of the assay. The SVC-AuNPs assay could detect SVCV-RNA as far as the 10^–3^ TCID_50_ ml^ − 1^ dilution (Tube 8; blue color)

Histological observation followed by polymerase chain reaction (PCR) [39], or cohabitation studies as performed by El-Matbouli & Soliman [40] to demonstrate transmission of CyHV-3 virus from goldfish (*Carassius auratus auratus*) to naïve carp have been the classical method of detection and demonstration of transmission of pathogen(s) to new hosts. PCR-based methods coupled to electron microscopy have also been used in categorizing aquatic pathogens, such as in the classification of the parasitic ciliate infecting shrimp [41]. However, updated methods for rapid detection is required to tackle the rapid spread of communicable pathogens in aquatic farming, especially in densely populated environment used in aquaculture. For example, preliminary differentiation of CyHV-3 from channel catfish virus was performed by restriction analysis of purified DNA extracts and led to a PCR-based method detection [42, 43]. Subsequently, a 1 step process was developed using loop-mediated isothermal amplification (LAMP) without requiring a thermal cycler for detection of CyHV-3 [44–46]. Alternatively, nested PCR or the capture of viral particles by antibodies followed by LAMP can also be used for highly sensitive detection of CyHV-3 [47–49]. PCR based methods of detection can be coupled with DNA-array technology for rapid detection of secondary infections in diseased fish [50]. For rapid and visual based detection for CyHV-3, the product of LAMP-PCR is visualized by mixing with SYBR-Green I to confirm infection [51]. Attachment of single stranded DNA molecules to gold nanoparticles allows for rapid (15 min) and sensitive detection (10^− 3^ TCID_50_ ml^− 1^) of SVCV RNA based on visualization (Fig. 2; reproduced from Saleh et al. [52] with permission from Springer Nature) of colloidal solution [53] and the procedure can be readily adapted for detection of aquatic viruses [54, 55]. In addition to the various molecular based methods, immunohistochemistry in terms of histological assays [56] or enzyme-linked immunosorbent assay (ELISA) are also used to detect viral infections [57–63]. Detection of viral particles in affected species and carriers is important to combat the spread of outbreaks [64]. Recently, a method described as liquid chip which combines flow cytometry, nanometer fluorescent microspheres with traditional chemical luminescence technology has been described for rapid detection of several *Rhabdoviridae* members including SVCV, IHNV and VHSV [65].

### Characterization

Traditionally, pathogens of aquatic organisms have been characterized by sequence analysis [66–69], and by microscopic examination of the pathogen and/or host by differing methods that include histological staining [70] and electron microscopy [71]. Recently, deep sequencing along with microarray hybridization has been used to identify pathogen microRNAs (miR) involved in gene regulation [72]. Similarly, real-time quantitative reverse transcription PCR (RT-qPCR) has been used to show differential expression of the host miR-10a-3p, a component of the master transcriptional regulator for spatial patterning genes termed homeobox (Hox) genes, during infections by VHSV rock bream iridovirus (RBIV) of olive flounder and rock bream [73]. Real-time quantitative PCR (qPCR) or deep RNA sequencing can be used in transcriptome analysis to characterize host-pathogen interactions [74]. Histological assays can be coupled with immunofluorescence techniques to further elaborate the ultrastructure make up of disease causing pathogens [75]. For example, indirect fluorescence immunohistochemistry was adapted to *E. ictaluri* using the monoclonal antibody Ed9 [76] as the primary antibody and a fluorescein isothiocyanate (FITC) labeled goat anti-mouse secondary antibodies [77]. This allowed identification of the bacteria with the tissue and contributed in demonstrating the role of abrasion sites as a route of infection for *E. ictaluri* in *I. punctatus* [78]. Reverse genetic experimentation in which the reading frame of various viral proteins are altered, such as the non-virion (NV) non-structural protein, G and M proteins, or recombinant viruses are produced has been used to investigate virulent factors in VHSV [79–83].

Several approaches have been applied to investigate the virulence mechanisms of bacterial pathogens. For example, in vivo induced antigen technology (IVIAT) relies on harvesting antibodies from host exposed to the pathogen of interest. The antibodies are then adsorbed against an in vitro culture of the pathogen, therefore removing the antibodies that react against antigens expressed in vitro. The only remaining antibodies, recognizing antigens specifically expressed in vivo, are then used to screen an expression library expressing random sequences from the pathogen genomes [84, 85]. Regarding aquatic species, IVIAT has been used to investigate *Edwardsiella tarda* [86], *Vibrio anguillarum* [87] as well as *A. salmonicida* subsp. *salmonicida* [88, 89] and, more recently, *Photobacterium damselae* subsp. *piscicida* [90].

Gene expression profiling has been applied to *I. multifiliis* to identify genes that are differentially regulated during the different life stages of the parasite [91, 92]. This approach has led to the discovery that gene expression in *I. multifillis* is extremely stage specific [91] and has led to a better understanding of the expression of the virulence genes in this parasite. Moreover, it has been reported that senescence of the parasite was correlated to a lower expression for the genes of its *Rickettsia* endosymbiont [92]. Similarly, Mai et al. performed an immunoproteomic analysis of *C. irritans* [93] using 2D gels and anti-*C. irritans* antibodies isolated from both rabbit and grouper (*Epinephelus coioides*). This approach allowed to identify several proteins that were differentially regulated between life stages of the parasite, including several antigenic ones with potential in vaccine development. Among the isolation were proteins of the cytoskeletal apparatus (β-tubulin and actin), as well as the enzyme enolase and the heat shock protein hsp70 [93]. Moreover, Yin et al. [94] conducted an analysis of the transcriptomes of trophont, either untreated or treated at either 12 or 25 °C. The authors described up-regulation of several genes required for the cell’s survival at lower temperature and entry into dormancy [94].

### Proteomics

Exogenous expression in bacteria of proteins found in aquatic pathogens followed by PAGE-Gel analysis and Western blotting with native proteins can be used to detect in vivo posttranslational modifications by differences in observed mass [95, 96]. Monoclonal antibodies against CyHV-3 have been used to measure the expression kinetics of various proteins involved in protein assembly [97]. Analyses of whole proteomes have also been carried out; for example, in *Y. ruckeri* [98], where the proteomes of four different isolates were compared under iron-replete and iron-depleted conditions. This revealed the spread of the bacterium’s response to iron depletion as 61 proteins were found to be differentially expressed (35 up and 26 down-regulated). Among these were several siderophores (iron acquisition molecules that play a crucial role in the microbial infection process when iron is often the bacteria’s limiting factor) that were up-regulated and catalase that were down-regulated. Interestingly, the bacteria appeared to shift from iron-based superoxide dismutase (SodB, 28.6 fold downregulated) to manganese-based using a manganese based superoxide dismutase (SodA) that was 5.6-fold upregulated.

Differential transcriptional patterns obtained from qPCR based methods [99, 100] can be coupled to protein-based studies [101] to enhance our understanding of pathogen-host interactions. For protein purification, antibodies raised against the pathogen(s) of interest can be used to capture proteins involved in host-pathogen interactions which are subsequently identified by mass spectrometry [102]. Such studies have been used to differentiate between host pathogen interactions of susceptible carp versus carrier goldfish for CyHV-3 entry and replication [103, 104]. Additionally, proteomic based approach revealed that although all 156 open reading frames (ORFs) are CyHV-3 are transcribed during viral maturation [105], only 46 proteins are incorporated into mature virions [106, 107].Whereas, exogenous expression of viral proteins can demonstrate lethal properties for those proteins [108]. Therefore, proteomic should be used to enhance and elucidate transcriptional based data.

## Treatment

### RNA interference (RNAi)

RNA mediated interference (RNAi) machinery is presumed to have developed as a defensive mechanism in eukaryotic organisms against viruses and transposable elements [109]. RNA-mediated interference (RNAi) by the use of short double-stranded RNA (dsRNA) was originally demonstrated in *Caenorhabditis elegans* by Fire et al. [110], and the mechanism and machinery for the function of small non-coding RNA in RNAi has since been worked out in great detail for a variety of organisms [111, 112]. During the post-transcriptional gene silencing (PTGS) of exogenous transcripts by RNAi, the RNA-induced silencing complex (RISC) converts long dsRNA transcripts into siRNA oligos (21-25 nt), which guide the complex by antisense complementation to degrade targeted genes [113]. RNAi technology has been important in understanding gene function in aquatic diseases [114] and can be used to study RNA-based viruses which are traditionally investigated by reverse genetics [115]. RNAi-based approaches are also suitable for the development of novel therapies against viral diseases of livestock and aquatic organisms and represent a promising method in developing novel therapeutics and antiviral medications [116]. A limited number of studies have reported about the treatment of viral diseases by RNAi, although RNAi based therapies for viral diseases have been in the pipeline to treat invertebrate, vertebrate and even human pathogens [117].

### Studies that used RNAi technology in aquatic medicine

#### Viral

In a recent study, feeding shrimp with bacteria coding for dsRNA that targeted endogenous shrimp non-essential Rab7 and STAT genes caused systemic induction of the RNAi pathway against the targeted genes [118]. The technique has been applied to provide protection from an important disease in shrimp farms, termed White spot syndrome virus (WSSV), by feeding shrimp with bacteria expressing dsRNA against several important viral genes [119]. Inhibition by RNAi of WSSV was first demonstrated in a non-Shrimp cell line, termed SISK, and by intramuscular injection of live shrimp [120, 121], and the results were verified when shrimp were fed with dsRNA-transcribing bacteria [122]. Permanent *epithelioma papulosum cyprini* (EPC) and *chinook salmon embryonic* (CHSE-214) fish cell lines that express long dsRNA which target the G protein of viral hemorrhagic septicemia virus (VHSV) inhibited in vitro replication of VHSV without stimulating the interferon pathway [123]. Treatment with formaldehyde-attenuated bacterial cells that produce dsRNA targeting the hemagglutinin gene of ISAV inhibited in vitro viral replication [124]. RNAi targeting the nucleoprotein ‘N’ or phosphoprotein “P” has been shown to inhibit in vitro replication of SVCV [125]. Similarly, RNAi experiments targeting thymidine kinase ‘TK’ or DNA polymerase ‘DP’ of CyHV-3 inhibited in vitro replication [126]. CyHV-3 is most effectively inhibited by RNAi when multiple viral genes are targeted [126, 127].

#### Bacterial/parasitic

RNAi treatment has shown promising results in the treatment of parasitic infections in fish. Saleh et al. [128] demonstrated that in vitro RNAi knockdown of ATP/ADP antiporter and methionine aminopeptidase II of the *Heterosporis saurida*, parasite of the lizardfish (*Saurida undosquamis)*, reduced targeted gene transcription and spore counts in cell culture assays. Potential for RNAi-based medicine has been demonstrated in vivo by treating the oligochaete host *Tubifex tubifex* for the cnidarian myxozoan parasite (*Myxobolus cerebralis*) which causes whirling diseases in salmonid fish [129]. In subsequent trials, Sarker et al. [130] showed that *T. tubifex* soaked in solution containing dsRNA targeting the serine protease of the *M. cerebralis* inoculated the cnidarian myxozoan parasite from infecting the rainbow trout (*Oncorhynchus mykiss) host.*

### Is CRISPR/Cas based medicine on the horizon?

Cre (causes recombination) and other tyrosine recombinases have traditionally been used for genomic editing. These tyrosine site-specific recombinases are typically used to reintegrate exogenous DNA flanked by palindromic into a host genome such as observed in the Cre/Lox system in which Cre is used with the accompanying Lox palindromic DNA sequence [131]. A newer genome editing tool termed CRISPR/Cas (clustered regularly interspaced short palindromic repeats/CRISPR-associated), takes advantage of the prokaryotic and archaea immune system to reintegrate foreign DNA using a Cas protein and a guide RNA (gRNA) [132]. In addition to gene editing, the nuclease activity of the CRISPR/Cas pathway can be induced to degrade foreign RNA/DNA [133]. The CRISPR/Cas gene suppression technique was recently used by Zhao et al. [134] to confirm that RNAi knockdown of CyHV-3 TK and DP genes reduced viral replication and virus titer as reported previously by our group [126].

## Conclusion

Prophylactic treatments, for example aiming at strengthening or preparing the immune response such as vaccination, are always preferable to therapeutic ones [135–138]. However, preventative therapies are not always possible or practical, therapies based on small nucleotide based medicine such as siRNA or CRISPR/Cas are on the horizon. RNAi-based technology has already been suggested to be useful in aquatic and fish medicine to combat various types of diseases caused by viral and parasitic agents [116, 139]. Effectiveness of using RNAi or other nucleic based therapies rely on targeting pathogens transcripts that interfere with the hosts defensive capabilities used in viral entry or replication [140, 141]. RNAi and CRISPR/Cas mediated interference [133] along with the use small molecules to promote endogenous host response to viral infections [142, 143] are powerful emerging therapy strategies to deal with diseases in aquatic medicine. For those interesting cases where some aqua-species have become invasive, using a disease may be the methodology used to control the threat. For example, the release of CyHV-3 is seriously being considered to eradicate the invasive carp in Australia to restore populations of native fish species [144, 145] following the incidental example happening in the USA [146].

## Abbreviations

CHSE-214: *Chinook salmon embryonic*
Cre: Causes recombination
CRISPR/Cas: Clustered Regularly Interspaced Short Palindromic Repeats/CRISPR associated protein 9
CyHV-3: Cyprinid herpesvirus 3
EPC: *Epithelioma papulosum cyprini*
FITC: Fluorescein isothiocyanate
gRNA: Guide RNA
Hox: Homeobox
IHNV: Infectious hematopoietic necrosis virus
ISAV: Infectious salmon anaemia virus
IVIAT: In vivo induced antigen technology
KHVD: Koi herpesvirus disease
LAMP-PCR: Loop mediated isothermal amplification PCR
NV: Non-virion protein
PAGE-Gel: Polyacrylamide gel electrophoresis gel
PCR: Polymerase chain reaction
qPCR: Real-time quantitative (RT) PCR
RBIV: Rock bream iridovirus
RNAi: RNA interference
RSIV: Red sea bream iridovirus (RSIV)
siRNA: Small inhibitory Ribonucleic acid
SVCV: Spring viremia of carp virus
VHSV: Hemorrhagic septicemia virus
WSSV: White Spot Syndrome Virus
